# Facile hydrothermal synthesis of layered 1T′ MoTe_2_ nanotubes as robust hydrogen evolution electrocatalysts

**DOI:** 10.3389/fchem.2022.1005782

**Published:** 2022-09-27

**Authors:** Yuxi Lei, Xuefeng Xiao, Tianpeng Ma, Weiyin Li, Huan Zhang, Chao Ma

**Affiliations:** ^1^ School of Electrical and Information Engineering, North Minzu University, Yinchuan, China; ^2^ The Key Laboratory of Physics and Photoelectric Information Functional Materials, North Minzu University, Yinchuan, China

**Keywords:** MoTe_2_ nanotubes, layered nanostructure, electrocatalysts, hydrogen evolution reaction, hydrothermal method

## Abstract

Layered transition metal dichalcogenides (TMDs), such as molybdenum ditelluride (MoTe_2_), have attracted much attention because of their novel structure-related physicochemical properties. In particular, semi-metallic-phase MoTe_2_ (1T′) is considered as a competitive candidate for low-cost electrocatalysts for water splitting. However, there are few reports on the simple hydrothermal synthesis of MoTe_2_ nanostructures compared with other layered TMDs. In this study, a facile one-step hydrothermal process was developed for the fabrication of layered MoTe_2_, in which uniform nanotubes with a few layers of 1T′ MoTe_2_ were fabricated at a lower temperature for the first time. The as-obtained MoTe_2_ nanotubes were fully characterized using different techniques, which revealed their structure and indicated the presence of layered 1T′ nanocrystals. The efficient activity of MoTe_2_ nanotubes for the electrocatalytic hydrogen evolution reaction (HER) in 0.5 M H_2_SO_4_ was demonstrated by the small Tafel slope of 54 mV/dec^−1^ and endurable ability, which is attributed to the abundant active sites and remarkable conductivity of 1T′ MoTe_2_ with a few-layer feature. This provides a facile method for the design and construction of efficient layered MoTe_2_ based electrocatalysts.

## 1 Introduction

Layered transition metal dichalcogenides (TMDs) have attracted considerable attention in recent decades because of their novel structures and fascinating physical and chemical properties (Mas-Balleste et al., 2011; Xu et al., 2013; Tan et al., 2017; Ji and Choi, 2022). The layered structure consists of a transition metal atom sheet sandwiched between two sheets of chalcogen atoms, such as *MX*_2_ (*M* = W, Mo and *X* = S, Se, Te) (Qian et al., 2014; Sun et al., 2015), which has broad prospects for applications in optoelectronics (Khan and Leuenberger, 2018), photodetection (Malik et al., 2022), nanoelectronics (Wang et al., 2012) and electrochemistry (Chia et al., 2015), and has been well-studied in WS_2_, MoS_2_ and MoSe_2_ based 2D nanostructures in recent years (Bonaccorso et al., 2015; Zhang et al., 2016; Manzeli et al., 2017; Jha et al., 2019; Han et al., 2020; Singh et al., 2022a; Singh et al., 2022b). In particular, with the demand for effective catalysts for clean energy hydrogen production, TMDs have been widely studied and are considered to be as one of the most outstanding electrocatalysts for the hydrogen evolution reaction (HER) (Jin et al., 2018; Fu et al., 2021; Zhang et al., 2021; Li et al., 2022). TMDs nanostructures possess the capability of tuning catalytic active sites in comparison with their bulk counterparts. Importantly, they represent earth-abundant and low-cost alternatives to noble metal Pt groups (Chandrasekaran et al., 2019; Guo et al., 2019; Li et al., 2021).

Recently, van der Waals (vdW) layered TMDs MoTe_2_ has been widely reported for its potential HER performance with enhanced activity (Jinbong et al., 2017; McGlynn et al., 2018; Qiao et al., 2018; Bhat and Nagaraja, 2019; McGlynn et al., 2019; Zazpe et al., 2021). MoTe_2_ has two typical stable phases: a hexagonal structure and a distorted monoclinic structure, also known as 2H- and 1T′-, respectively (Manchanda et al., 2020; Singh et al., 2020). 1T′-MoTe_2_ exhibits semi-metallic characteristics and high conductivity, which favor efficient electrocatalytic activity. Seok et al. investigated the enhanced HER driving Peierls-type lattice distortion in 1T′-MoTe_2_ single crystals prepared using a solid-state method (Jinbong et al., 2017). McGlynn et al. studied the rapid electrochemical activation of 1T′-MoTe_2_ nanocrystals for HER, which originates from the adsorption of H onto Te sites on the surface of 1T′-MoTe_2_ (McGlynn et al., 2019). Mao et al. (2021) synthesized MoTe_2_ nanowires using a mesoporous silica SBA-15 template *via* a nanocasting method and investigated their HER performances. Besides, Lu et al. (2020) prepared 1T′ MoTe_2_ on carbon cloth *via* direct vapor deposition and demonstrated enhanced and stable HER performance in 1 M H_2_SO_4_. Zazpe et al. (2021) grew 2D flaky MoTe_2_ nanosheets on TiO_2_ nanotubes *via* atomic layer deposition for excellent pollutant photodegradation and HER properties. The aforementioned MoTe_2_ catalysts were prepared mainly using a high-temperature solid-state method or chemical vapor deposition. Sun et al. (2016) synthesized 1T′-MoTe_2_ nanoflowers using a solution method at a relatively low temperature of 300°C. Liu et al. (2017) prepared 1T′-MoTe_2_ nanosheets using a colloidal chemical strategy at 320°C. However, the synthesis of MoTe_2_ nanostructures with layered ultrathin sheets using a simple hydrothermal process at low temperatures has rarely been reported.

In this study, we prepared MoTe_2_ nanotubes with ultrathin nanosheets by a facile one-step hydrothermal process for the first time. The growth of MoTe_2_ nanosheets was derived from a template of Te nanorods, which were reduced rapidly from the TeO_2_ precursor *via* a moderate reductant of ascorbic acid, forming a tubular hierarchical structure with a diameter of 200 nm. The MoTe_2_ nanotubes demonstrated efficient and stable HER performance, exhibiting a lower overpotential of −374 mV at −100 mA·cm^−2^ and a Tafel slope of 54 mV dec^−1^, which are better than those of another typical nanostructure of MoTe_2_ nanoparticles. The improved electrocatalytic activity originates from the effective active sites in the nanosheet structure and high conductivity of 1T′-MoTe_2_. This facile synthetic strategy is expected to be helpful in the design of novel MoTe_2_-based electrocatalysts with 3D hierarchical nanostructures.

## 2 Experimental section

### 2.1 Synthesis of MoTe_2_ nanostructures

The MoTe_2_ nanotubes were synthesized *via* a one-step hydrothermal method using the following procedure. Tellurium dioxide (8 mmol, TeO_2_, 5N, Aladdin Reagent) was added to a 100 ml transparent solution containing 1.2 g ascorbic acid (C_6_H_8_O_6_, 99%, Sinopharm), and 40 ml ethanolamine (C_2_H_7_NO, 99%, Macklin Reagent) was added to the above suspension under constant stirring to form a light-yellow transparent solution. The configured solution was transferred to a 200 ml Hastelloy autoclave and mixed with 4 mmol molybdenum hexacarbonyl [Mo(CO)_6_, 98%, Macklin Reagent] that was directly added. The autoclave was heated to 180°C with a rate of 3°C/min and kept 20 h under constant stirring at 200 rpm. After the reaction, the autoclave was naturally cooled to room temperature, and the black precipitate was collected and washed three times with deionized water and ethanol. Finally, the product was desiccated in an oven at 60°C. The experimental process was schematic illustrated by Supplementary Figure S1.

For comparison, another MoTe_2_ nanostructure was prepared. MoTe_2_ nanoparticles were prepared using a similar procedure but with a different Te source without the addition of ascorbic acid. Te powder (2 mmol, Te, 5N, Aladdin Reagent) and 1 mmol Mo(CO)_6_ were directly added to a 200 ml Hastelloy autoclave containing 110 ml deionized water under constant stirring, and then 10 ml hydrazine hydrate aqueous solution (H_4_N_2_·*x*H_2_O, 85%, Sinopharm) was dropped into the above suspension. The autoclave was heated to 200°C at a rate of 3°C/min and maintained at 200 rpm for 20 h with constant stirring. The second step was the same as that used for the MoTe_2_ nanotubes.

### 2.2 Characterizations

The phase and crystal structures of the as-prepared samples were analyzed using X-ray powder diffraction (XRD) patterns measured on an X-ray diffractometer (Rigaku SmartlabSE) with Cu Kα radiation (*λ* = 1.5406 Å) at a scanning rate of 8°/min in the 2θ range of 10°C–80°C. The morphologies of the samples were observed using field-emission scanning electron microscopy (FESEM, ZEISS SIGMA 500) at an accelerating voltage of 10 kV. The crystalline characteristics of the MoTe_2_ nanostructure, dark-field scanning transmission electron microscopy (STEM), and energy-dispersive X-ray spectroscopy (EDX) mapping were performed using field-emission transmission electron microscopy (FETEM, JEOL JEM 2100F, 200 kV). X-ray photoelectron spectroscopy (XPS) was performed on an ESCALAB Xi+ X-ray photoelectron spectrometer (Thermo Fisher Scientific Ltd.) using Al-Kα radiation. The Raman spectrum was collected by Raman spectroscopy (Horiba Ltd.) using a 532 nm laser. The Brunauer-Emmett-Teller (BET) method was used to calculate the specific surface areas of the samples *via* N_2_ adsorption-desorption isotherms using a Micromeritics ASAP 2460 instrument.

### 2.3 Electrochemical measurements

All electrochemical measurements were carried out using an electrochemical workstation (CHI650E, Shanghai CH Instruments) with a three-electrode system in 0.5 M H_2_SO_4_ (unless otherwise stated). A graphite rod and Ag/AgCl were used as counter and reference electrodes, respectively. For the working electrode, the samples (5 mg) were added to 1 ml of isopropanol containing 0.25% Nafion under ultrasonication for 1 h, and 20 μl of the ink was coated onto the L-type glassy carbon electrode (5 mm in diameter) and dried naturally. All the potentials were converted to the reversible hydrogen electrode (RHE) using the following equation: *E* (vs. RHE) = *E* (vs. ref) + (0.059 V) × pH_electrolyte_ + *E*_0_ (ref vs. SHE), where *E*_0_ (ref vs. SHE) is the standard potential of the reference electrode versus the standard hydrogen electrode (SHE). Linear sweep voltammetry (LSV) curves were obtained in the selected potential range at a scan rate of 5 mV/s without iR compensation. The Tafel slope was fitted using the equation *η* = *a* + *b*log*j*, where *η* is the overpotential, *a* is a constant and *b* is the Tafel slope. Cyclic voltammetry (CV) data were collected at scan rates of 10 mV/s, 20 mV/s, 40 mV/s, 60 mV/s, 80 mV/s, and 100 mV/s in selected potential ranges to estimate the electrochemical active surface area (ECSA) and double-layer capacitance (*C*_dl_). Electrochemical impedance spectra (EIS) were obtained in the frequency range of 0.1 Hz–10^6^ Hz with an amplitude of 10 mV. Chronoamperometry was performed for 20 h at a potential of −0.2 V (vs. RHE)

## 3 Results and discussion

### 3.1 Morphology and phase

**FIGURE 1:**
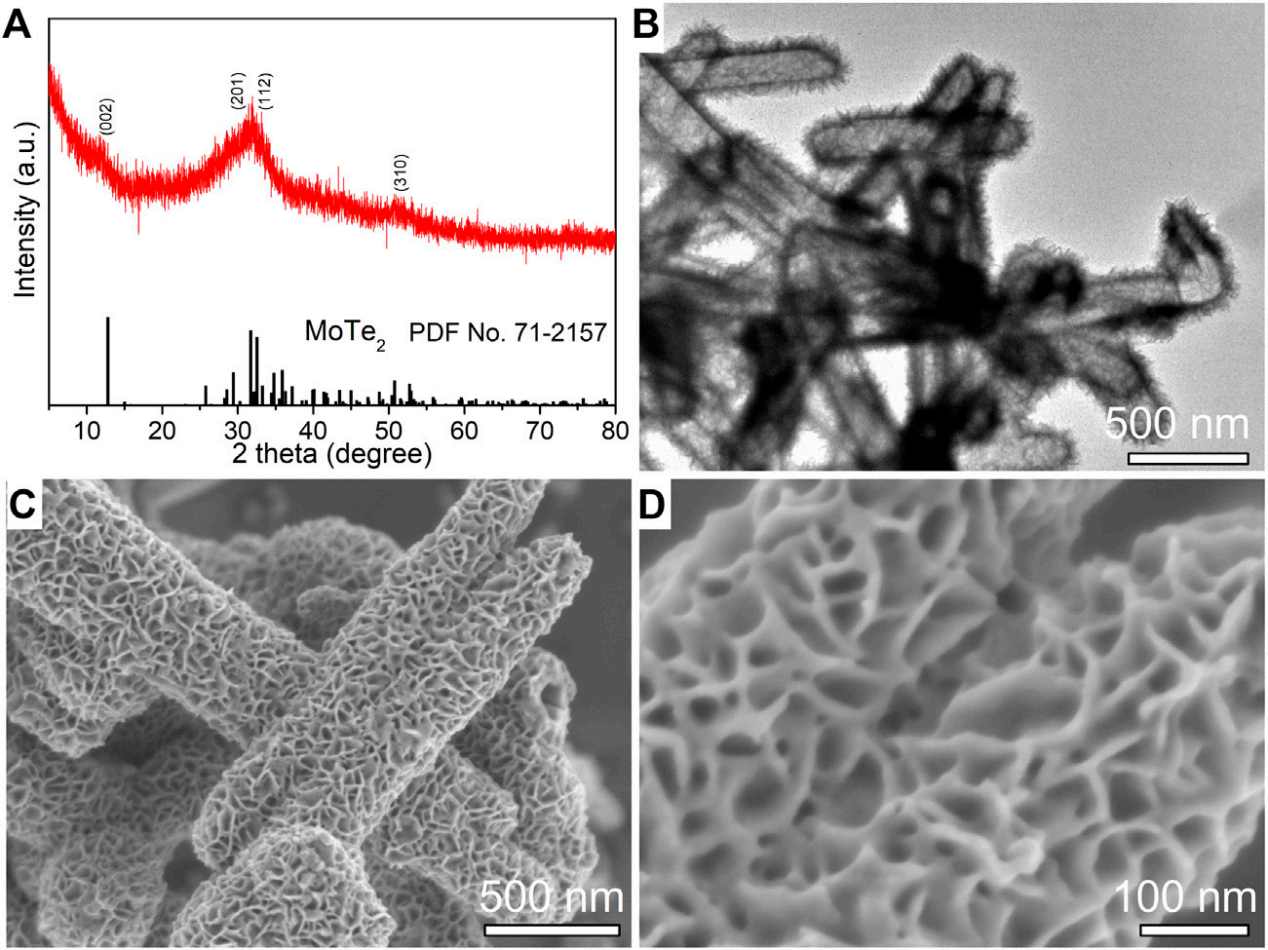
XRD pattern **(A)**, a typical TEM image **(B)**, low-resolution **(C)** and high-resolution **(D)** SEM images of as-synthesized MoTe_2_ nanotubes.

The phase and morphology of the as-synthesized MoTe_2_ powder obtained by a facile ascorbic acid (VC)-assisted hydrothermal method were characterized by XRD, SEM, and TEM. Figure 1A shows the collected XRD pattern, the diffraction peaks at 12°C, 29°C, 32°C, and 51°C match the (0 0 2), (2 0 1), (1 1 2), and (3 1 0) planes of 1T′ MoTe_2_ (JCPDS No. 71–2,157), and the low intensity and broadness of the diffraction peak (0 0 2) are related to a few sheets of the MoTe_2_ nanostructure and poor crystallinity by the hydrothermal route, which is similar to that of a few MoS_2_ nanosheets (Lei et al., 2017b; Liu et al., 2019). No other impurity peaks were detected, confirming that MoTe_2_ nanocrystals were synthesized. A typical TEM image (Figure 1B) indicates distinctly uniform tubular nanosheets on the microstructure of the products. A typical SEM image in Figure 1C and the corresponding high-resolution SEM image (Figure 1D) indicate that the nanotubes were decorated with ultrathin sheets on their surface, and the nanosheets interlaced with each other, forming a hierarchical structure. An individual nanotube with a diameter of approximately 200 nm and a length of several micrometers. Uniform MoTe_2_ nanotubes were synthesized using a facile hydrothermal method.

**FIGURE 2:**
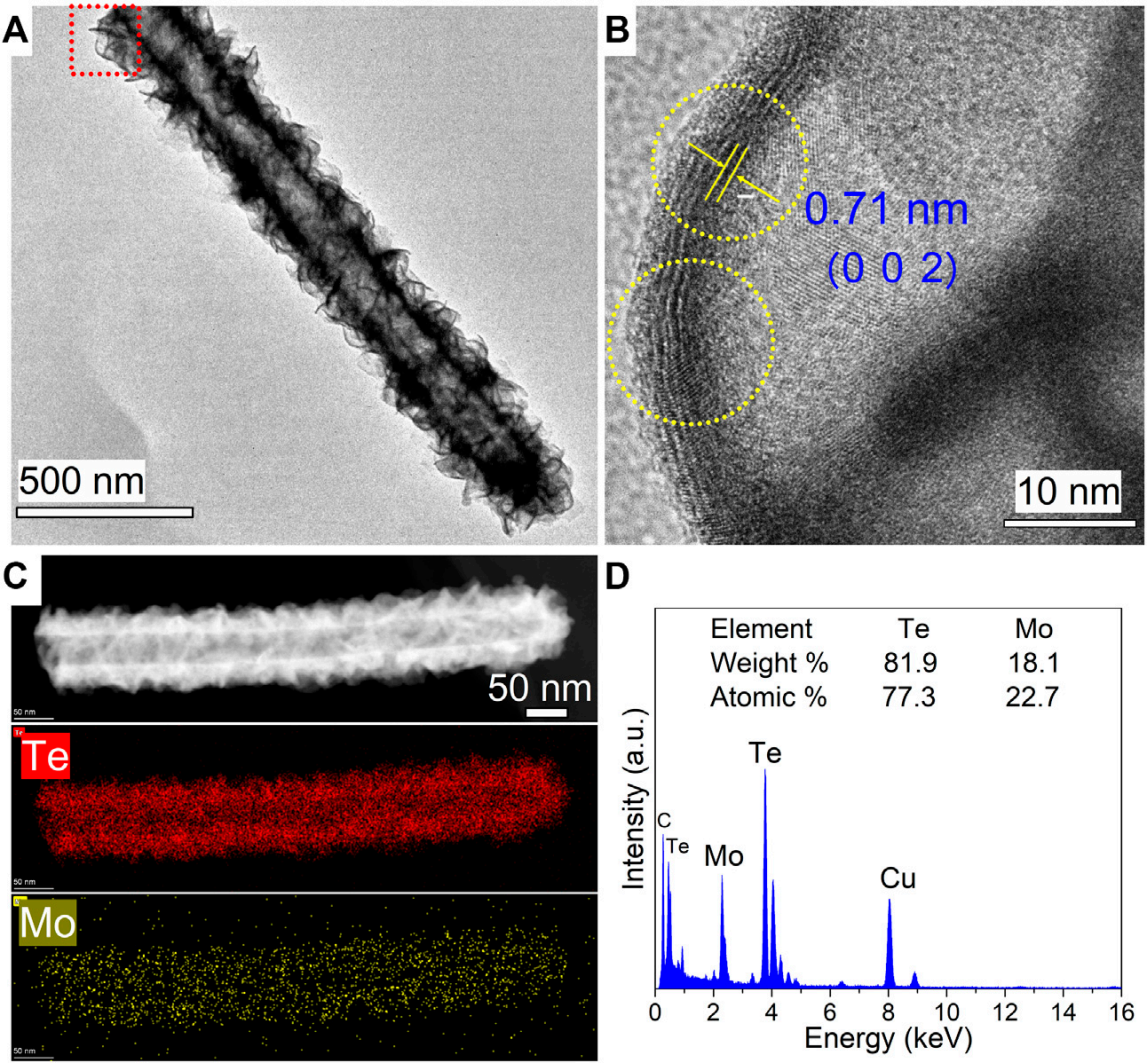
**(A)** Typical TEM image of an individual as-synthesized MoTe_2_ nanotube. **(B)** Corresponding HRTEM images in the red rectangular areas in **(A)**. **(C)** HAADF-STEM image and corresponding elemental mapping images of an individual MoTe_2_ nanotube. **(D)** The collected EDX spectrum.

The microstructural components and crystalline characteristics of the as-synthesized MoTe_2_ nanotubes were further investigated using HRTEM analysis. As shown in Figure 2A, an individual nanotube manifested a distinct tubular structure with a diameter of 200 nm and length of approximately 2 μm, on which thin sheets were uniformly distributed. The clear lattice stripes with a spacing of 0.71 nm are assigned to the (0 0 2) plane of the monoclinic MoTe_2_ crystal from the HRTEM image in Figure 2B, which is slightly larger than that of the standard MoTe_2_ crystal; the curved lattice fringe represents the characteristics of the layered TMDs nanosheets obtained by the wet chemical method (Lei et al., 2018; Wang et al., 2021, Zazpe et al., 2021). STEM-EDX mapping performed on a selected individual MoTe_2_ nanotube revealed a uniform and homogeneous elemental distribution of Te and Mo in the integrated MoTe_2_ nanotube (Figure 2C). The STEM-EDX spectrum and analytical results obtained for the entire nanotube are shown in Figure 2D, and are close to the stoichiometric proportion of the MoTe_2_ compound.

The growth of MoTe_2_ nanotubes using a facile hydrothermal method was explored using controlled experiments under different conditions. The additive agent VC plays a key role in the formation of uniform tubular MoTe_2_ nanosheets. VC can reduce TeO_2_ to Te nanorods in the precursor solution (Xi et al., 2006; Lei et al., 2017a) and act as a template/seed to grow MoTe_2_ nanosheets. Uniform MoTe_2_ nanotubes were obtained when an appropriate amount of VC was used (Figure 1; Supplementary Figure S2D); otherwise, irregular Te impurities and non-uniform MoTe_2_ microtubes with rough surfaces were obtained (Supplementary Figures S2,3).

**FIGURE 3:**
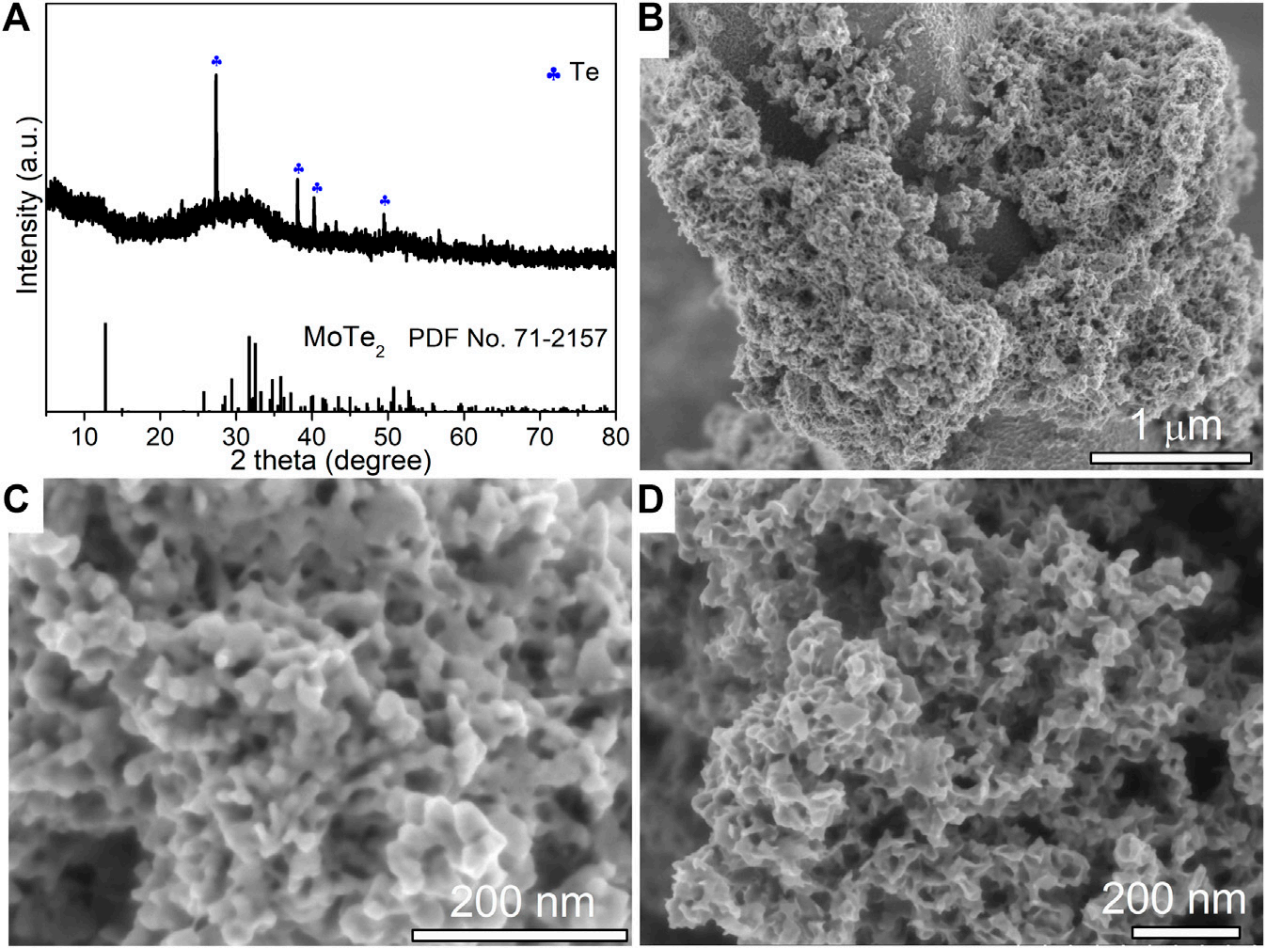
XRD pattern **(A)**, low-resolution SEM image **(B)** and high-resolution SEM images **(C,D)** of as-synthesized MoTe_2_ nanoparticles. The insets show the EDX analysis collected from the corresponding objects.

To investigate the facile hydrothermal route for the synthesis of MoTe_2_ nanocrystals, we attempted another process to prepare MoTe_2_ nanoparticles using Te powder as the Te source and hydrazine hydrate as the reducing reagent under different reaction conditions. Figure 3A shows the XRD pattern of the as-prepared sample, where the broad hump diffraction peaks around 12°C, 32°C, and 51°C are ascribed to the (0 0 2), (1 1 2), and (3 1 0) planes of 1T′ MoTe_2_ (JCPDS No. 71–2,157), respectively, which is consistent with the characteristics of MoTe_2_ nanotubes. Notably, a small amount of incompletely reacted Te impurities is detected. From the low-resolution SEM image (Figure 3B), it can be seen that irregular particles aggregated on a large rod (Te seed). The high-resolution SEM images in Figures 3C,D indicate that the size of the fine particles is approximately dozens of nanometers. In addition to the nanoparticle morphology, there are large quantities of amorphous sheets, forming a porous structure. The EDX spectrum and analytical results (insets of Figures 3C,D) collected from the corresponding objects are close to the stoichiometric ratio of MoTe_2_. Then, only irregular MoTe_2_ nanoparticles were obtained using the as-designed process but with uniform layered nanosheets.

### 3.2 XPS analysis

**FIGURE 4:**
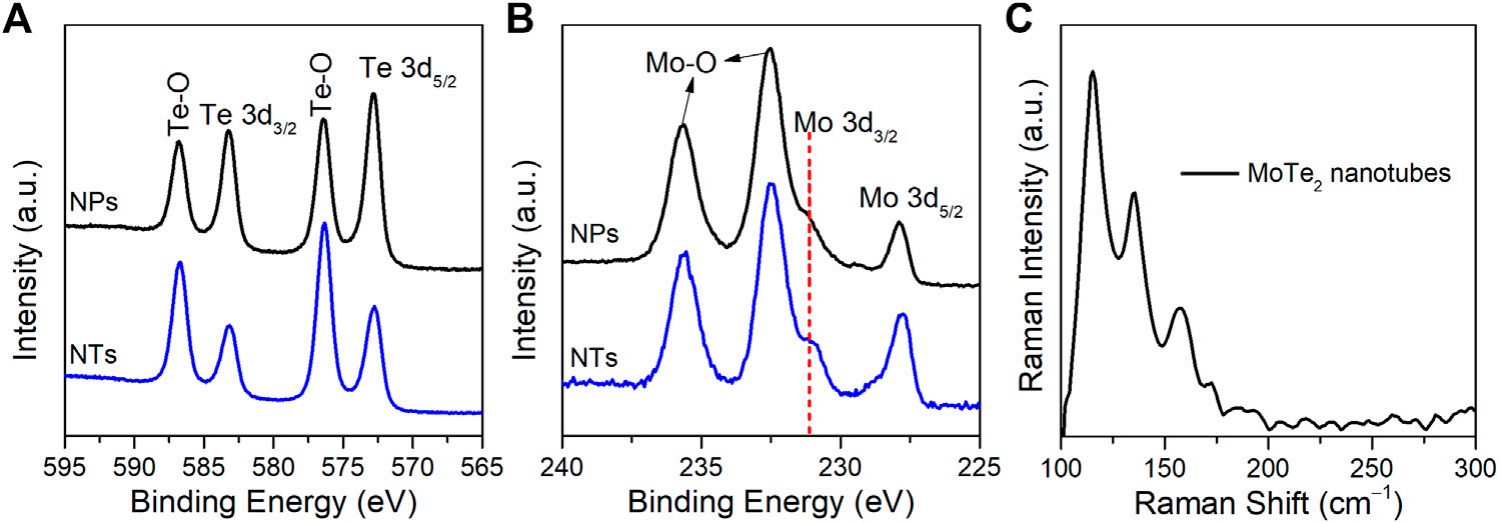
High-resolution XPS spectra of **(A)** Te 3d and **(B)** Mo 3d of MoTe_2_ nanotubes (NTs) and MoTe_2_ nanoparticles (NPs). **(C)** Raman spectrum of the as-synthesized MoTe_2_ nanotubes.

The elemental compositions and surface chemical states of the as-prepared MoTe_2_ nanocrystals were investigated using XPS. In the high-resolution Mo 3d spectrum, as illustrated in Figure 4A, the peaks at 572.8 eV and 583.2 eV correspond to Te 3d_5/2_ and Te 3d_3/2_, respectively, which are ascribed to the typical peaks of Te-Mo bonds (Guo and Wang, 2018; Li et al., 2020). The other two satellite peaks at 576.3 eV and 586.7 eV originate from the Te-O bond on sample exposed to ambient atmosphere, which is commonly detected in nanostructured tellurides (Luxa et al., 2017; Anantharaj et al., 2018). As for the Mo 3d (Figure 4B), the peaks at 227.8 and 231.1 eV should be ascribed to Mo 3d_5/2_ and Mo 3d_3/2_, similarly two observed peaks at 232.5 eV and 235.6 eV related to Mo-O bonds due to dynamic surface states. These findings are consistent with those of previous studies (Zazpe et al., 2021; Wang et al., 2021). Figure 4C shows the Raman spectrum of the as-synthesized MoTe_2_ nanotubes under a 532 nm laser, where the peaks situated at 115 cm^−1^, 135 cm^−1^, and 158 cm^−1^ correspond to the A_g_ modes. Notably, the absence of a peak at approximately 230 cm^−1^ which is the phase-specific Raman peak of 2H-MoTe_2_, indicating 1T′-MoTe_2_ was obtained using a facile solution-phase synthetic method.

The nitrogen adsorption–desorption isotherm was employed to calculate the specific surface area of the samples using the BET method, as shown in Supplementary Figure S4, which can be identified as type IV with hysteresis loops, confirming typical nanocrystalline structures. The specific area of the MoTe_2_ nanoparticles was approximately 87 m^2^/g, which is significantly larger than that of the MoTe_2_ nanotubes, possibly because of its fine particles and mesoporous structure.

### 3.3 Electrochemical performance

**FIGURE 5:**
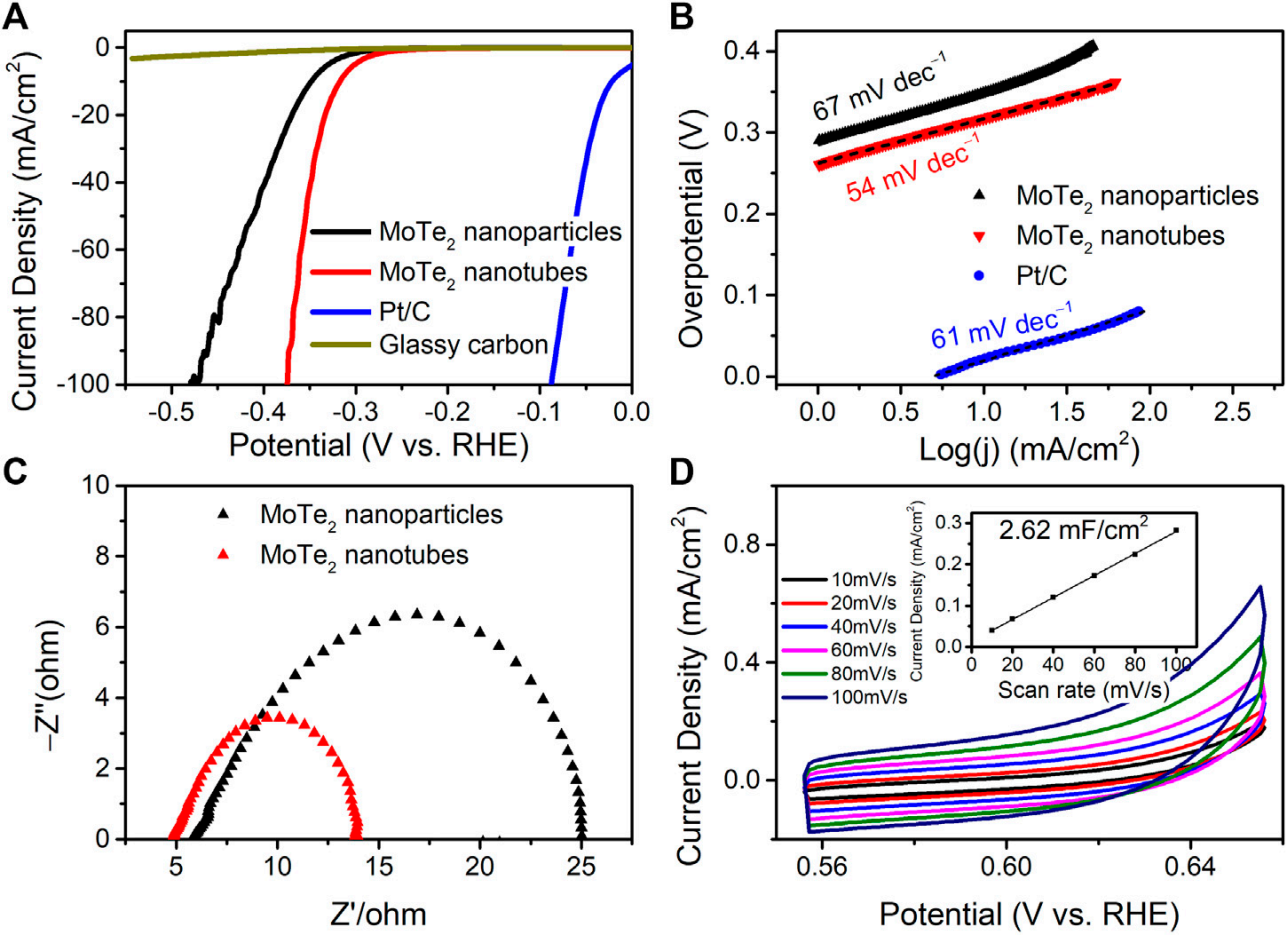
**(A)** LSV curves, **(B)** Tafel plots, **(C)** Nyquist plots, and **(D)** CV curves and linear fitting of the capacitive currents vs. CV scan rates (the inset) of the as-prepared MoTe_2_ nanocrystals for HER in 0.5 M H_2_SO_4_.

The electrocatalytic activity of the as-prepared MoTe_2_ nanocrystals for the hydrogen evolution reaction (HER) was investigated using electrochemical measurements and compared with that of commercial Pt/C. Figure 5A shows the linear sweep voltammetry (LSV) curves in a 0.5 M H_2_SO_4_ electrolyte. The as-prepared MoTe_2_ nanotubes exhibited lower overpotentials of −317 mV and −349 mV at current densities of −10 mA·cm^−2^ and −100 mA·cm^−2^, respectively, which were much smaller than those of MoTe_2_ nanoparticles and MoTe_2_ nanowires (Mao et al., 2021). The MoTe_2_ nanotubes obtained with different amounts of VC (except for 0 VC) showed similar catalytic performance (Supplementary Figure S5). It can be seen from the LSV curve that the current density increased rapidly with increasing potential in the MoTe_2_ nanotubes. The HER performance of MoTe_2_ related electrocatalysts reported in recent literature is listed in Supplementary Table S1.

Tafel plots and their slopes were obtained using the Tafel equation for the kinetic investigation of HER, as shown in Figure 5B. The Tafel slope of the MoTe_2_ nanotubes was 54 mV∙dec^−1^, which was even lower than that of the commercial Pt/C catalyst (61 mV∙dec^−1^). A slight decrease in the Tafel slope (67 mV∙dec^−1^) was observed for MoTe_2_ nanoparticles. The HER kinetics of MoTe_2_ nanotubes can be inferred from the Volmer-Heyrovsky mechanism (Watzele et al., 2018). The catalytic kinetics were further revealed by Nyquist plots from the electrochemical impedance spectra (EIS) shown in Figure 5C. The results indicate that the MoTe_2_ nanotubes exhibit a small charge-transfer resistance (*R*_ct_) in comparison with that of the MoTe_2_ nanoparticles, which is consistent with the LSV results. The electrochemical surface area (ECSA) of the MoTe_2_ nanotubes was further evaluated from the double-layer capacitances (*C*_dl_) measured by CV curves at different scan rates in the potential range of 0.55 V–0.65 V (Figure 5D). The *C*_dl_ of MoTe_2_ nanotubes is linear fitting to 2.62 mF∙cm^−1^.

**FIGURE 6:**
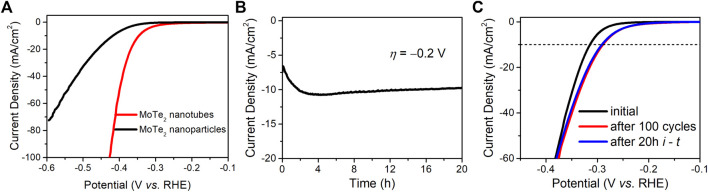
**(A)** LSV curves of the MoTe_2_ nanotubes and MoTe_2_ nanoparticles in 1.0 M H_2_SO_4_. Stability investigation of MoTe_2_ nanotubes for HER in 0.5 M H_2_SO_4_, **(B)** Chronoamperometry curves for 20 h and **(C)** LSV curves before and after 100 cycles as well as after 20 h Chronoamperometry test.

The endurance and long-term stability of the electrocatalysts were investigated. Figure 6A depicts the HER performance of the two MoTe_2_ nanocrystals exposed to a 1.0 M H_2_SO_4_ electrolyte. The MoTe_2_ nanotubes exhibited much lower overpotentials at a certain current density than MoTe_2_ nanoparticles. The potential slightly increased with the current density in the MoTe_2_ nanotubes, indicating an endurable stability of the HER activity even in a strong alkaline electrolyte. Figure 6B shows a curve of current density with time (*i* - *t*) under a fixed overpotential of −0.2 V by using chronopotentiometry measurements. It can be seen that the current density was distinctly increased in the first two hours, which is similar with recent reports about the gradual electrochemical activation of MoTe_2_ nanocrystals (McGlynn et al., 2019; Zazpe et al., 2021). Subsequently, the current density remained stable over time. Cyclic voltammetry measurements were conducted to assess stability. As shown in Figure 6C, the polarization curve of the MoTe_2_ nanotubes reveals a decrease in the overpotential (∼30 mV at a current density of −10 mA∙cm^−2^) after 100 cycles and a negligible change after 20 h of chronopotentiometry measurements, which is consistent with the *i*-*t* result. SEM and XPS were performed to evaluate the structural and chemical stability of the MoTe_2_ nanotubes (Supplementary Figure S6). The SEM images reveal no obvious changes in the morphological characteristics of the tubular nanosheets. No obvious change in the position of the peaks was observed in the XPS high-resolution Te 3d and Mo 3d spectra. Notably, there is an obvious decrease in the intensity of the Te-O peaks, suggesting that the electrochemical activation would originated from the Te sites of the layered MoTe_2_ nanosheets in the HER process, which is in line with previous reports (Zazpe et al., 2021; McGlynn et al., 2019).

## 4 Conclusion

In this work, two typical MoTe_2_ nanocrystals were prepared by low-temperature hydrothermal methods. Uniform MoTe_2_ nanotubes with hierarchical few-layer nanosheets were synthesized for the first time. An appropriate amount of VC contributed to the formation of tubular nanosheet structures. The as-synthesized MoTe_2_ nanotubes exhibited efficient performance in the hydrogen evolution reaction with a Tafel slope of 54 mV∙dec^−1^. A relatively gradual electrochemical activation and durable HER stability were also observed. The uniform nanosheet structure improved the electrochemical active sites and facilitated HER. The development of sheet-like MoTe_2_ nanocrystals using facile synthetic methods is expected to be helpful for the promising applications of layered MoTe_2_.

## Data Availability

The original contributions presented in the study are included in the article/Supplementary Material, further inquiries can be directed to the corresponding author.

## References

[B1] Anantharaj S. Karthick K. Kundu S. (2018). NiTe_2_ nanowire outperforms Pt/C in high-rate hydrogen evolution at extreme pH conditions. Inorg. Chem. 57, 3082–3096. 10.1021/acs.inorgchem.7b02947 29498515

[B2] Bhat K. S. Nagaraja H. S. (2019). Performance evaluation of molybdenum dichalcogenide (MoX_2_; X= S, Se, Te) nanostructures for hydrogen evolution reaction. Int. J. Hydrogen Energy 44, 17878–17886. 10.1016/j.ijhydene.2019.05.179

[B3] Bonaccorso F. Colombo L. Yu G. Stoller M. Tozzini V. Ferrari A. C. (2015). 2D materials. Graphene, related two-dimensional crystals, and hybrid systems for energy conversion and storage. Science 347, 1246501. 10.1126/science.1246501 25554791

[B4] Chandrasekaran S. Yao L. Deng L. Bowen C. Zhang Y. Chen S. (2019). Recent advances in metal sulfides: From controlled fabrication to electrocatalytic, photocatalytic and photoelectrochemical water splitting and beyond. Chem. Soc. Rev. 48, 4178–4280. 10.1039/c8cs00664d 31206105

[B5] Chia X. Eng A. Y. S. Ambrosi A. Tan S. M. Pumera M. (2015). Electrochemistry of nanostructured layered transition-metal dichalcogenides. Chem. Rev. 115, 11941–11966. 10.1021/acs.chemrev.5b00287 26426313

[B6] Fu Q. Han J. Wang X. Xu P. Yao T. Zhong J. (2021). 2D transition metal dichalcogenides: Design, modulation, and challenges in electrocatalysis. Adv. Mat. 33, 1907818. 10.1002/adma.201907818 PMC11468112 32578254

[B7] Guo Y. Park T. Yi J. W. Henzie J. Kim J. Wang Z. (2019). Nanoarchitectonics for transition-metal-sulfide-based electrocatalysts for water splitting. Adv. Mat. 31, 1807134. 10.1002/adma.201807134 30793387

[B8] Guo Z. Wang X. (2018). Atomic layer deposition of the metal pyrites FeS_2_, CoS_2_, and NiS_2_ . Angew. Chem. Int. Ed. 57, 5898–5902. 10.1002/anie.201803092 29607592

[B9] Han W. Liu Z. Pan Y. Guo G. Zou J. Xia Y. (2020). Designing champion nanostructures of tungsten dichalcogenides for electrocatalytic hydrogen evolution. Adv. Mat. 32, 2002584. 10.1002/adma.202002584 32491265

[B10] Jha R. K. D’Costa J. V. Sakhuja N. Bhat N. (2019). MoSe_2_ nanoflakes based chemiresistive sensors for ppb-level hydrogen sulfide gas detection. Sensors Actuators B Chem. 297, 126687. 10.1016/j.snb.2019.126687

[B11] Ji J. Choi J. H. (2022). Recent progress in 2D hybrid heterostructures from transition metal dichalcogenides and organic layers: Properties and applications in energy and optoelectronics fields. Nanoscale 14, 10648–10689. 10.1039/d2nr01358d 35839069

[B12] Jin H. Guo C. Liu X. Liu J. Vasileff A. Jiao Y. (2018). Emerging two-dimensional nanomaterials for electrocatalysis. Chem. Rev. 118, 6337–6408. 10.1021/acs.chemrev.7b00689 29552883

[B13] Jinbong S. Jun-Ho L. Suyeon C. Byungdo J. Hyo Won K. Min K. (2017). Active hydrogen evolution through lattice distortion in metallic MoTe_2_ . 2D Mat. 4, 025061. 10.1088/2053-1583/aa659d

[B14] Khan M. A. Leuenberger M. N. (2018). Optoelectronics with single layer group-VIB transition metal dichalcogenides. Nanophotonics 7, 1589–1600. 10.1515/nanoph-2018-0041

[B15] Lei Y.-X. Miao N.-X. Zhou J.-P. Hassan Q. U. Wang J.-Z. (2017a). Novel magnetic properties of CoTe nanorods and diversified CoTe_2_ nanostructures obtained at different NaOH concentrations. Sci. Technol. Adv. Mater. 18, 325–333. 10.1080/14686996.2017.1317218 28567178 PMC5439396

[B16] Lei Y. Wang J. Zhou J. Guo Z. Hassan Q. U. (2018). Fabrication and enhanced photocatalytic properties of novel 3D MoS_2_/Na_0.9_Mg_0.45_Ti_3.55_O_8_ heterostructures. Appl. Surf. Sci. 427, 733–741. 10.1016/j.apsusc.2017.08.169

[B17] Lei Y. Zhou J. Hassan Q. U. Wang J. (2017b). One-step synthesis of NiTe_2_ nanorods coated with few-layers MoS_2_ for enhancing photocatalytic activity. Nanotechnology 28, 495602. 10.1088/1361-6528/aa94ae 29048332

[B18] Li C. Liu M. Ding H. He L. Wang E. Wang B. (2020). A lightly Fe-doped (NiS_2_/MoS_2_)/carbon nanotube hybrid electrocatalyst film with laser-drilled micropores for stabilized overall water splitting and pH-universal hydrogen evolution reaction. J. Mat. Chem. A Mat. 8, 17527–17536. 10.1039/d0ta04586a

[B19] Li X.-Y. Zhu S.-J. Wang Y.-L. Lian T. Yang X.-Y. Ye C.-F. (2022). Synergistic regulation of S-vacancy of MoS_2_-based materials for highly efficient electrocatalytic hydrogen evolution. Front. Chem. 10, 915468. 10.3389/fchem.2022.915468 35755244 PMC9214220

[B20] Li Y. Yin Z. Cui M. Liu X. Xiong J. Chen S. (2021). Interface engineering of transitional metal sulfide–MoS_2_ heterostructure composites as effective electrocatalysts for water-splitting. J. Mat. Chem. A Mat. 9, 2070–2092. 10.1039/d0ta10815d

[B21] Liu M. Wang Z. Liu J. Wei G. Du J. Li Y. (2017). Synthesis of few-layer 1T'-MoTe_2_ ultrathin nanosheets for high-performance pseudocapacitors. J. Mat. Chem. A Mat. 5, 1035–1042. 10.1039/c6ta08206h

[B22] Liu Y. Li Y. Peng F. Lin Y. Yang S. Zhang S. (2019). 2H- and 1T- mixed phase few-layer MoS_2_ as a superior to Pt co-catalyst coated on TiO_2_ nanorod arrays for photocatalytic hydrogen evolution. Appl. Catal. B Environ. 241, 236–245. 10.1016/j.apcatb.2018.09.040

[B23] Lu D. Ren X. Ren L. Xue W. Liu S. Liu Y. (2020). Direct vapor deposition growth of 1T′ MoTe_2_ on carbon cloth for electrocatalytic hydrogen evolution. ACS Appl. Energy Mat. 3, 3212–3219. 10.1021/acsaem.9b01589

[B24] Luxa J. Voseck P. Maz Nek V. Sedmidubsk D. Pumera M. Lazar P. (2017). Layered transition-metal ditellurides in electrocatalytic applications—contrasting properties. ACS Catal. 7, 5706–5716. 10.1021/acscatal.7b02080

[B25] Malik M. Iqbal M. A. Choi J. R. Pham P. V. (2022). 2D materials for efficient photodetection: Overview, mechanisms, performance and UV-ir range applications. Front. Chem. 10, 905404. 10.3389/fchem.2022.905404 35668828 PMC9165695

[B26] Manchanda P. Kumar P. Dev P. (2020). Thickness dependence of hydrogen-induced phase transition in MoTe_2_ . Phys. Rev. B 101, 144104. 10.1103/physrevb.101.144104

[B27] Manzeli S. Ovchinnikov D. Pasquier D. Yazyev O. V. Kis A. (2017). 2D transition metal dichalcogenides. Nat. Rev. Mat. 2, 17033. 10.1038/natrevmats.2017.33

[B28] Mao J. Zhou L. Li Y. Tao Y. Chai K. Shi Y. (2021). Synthesis of MoTe_2_ nanowire as an efficient hydrogen evolution reaction material. Mater. Lett. 290, 129471. 10.1016/j.matlet.2021.129471

[B29] Mas-Balleste R. Gomez-Navarro C. Gomez-Herrero J. Zamora F. (2011). 2D materials: To graphene and beyond. Nanoscale 3, 20–30. 10.1039/c0nr00323a 20844797

[B30] Mcglynn J. C. Cascallana-Mat As I. Fraser J. P. Roger I. Mcallister J. Miras H. N. (2018). Molybdenum ditelluride rendered into an efficient and stable electrocatalyst for the hydrogen evolution reaction by polymorphic control. Energy Technol. 6, 345–350. 10.1002/ente.201700489

[B31] Mcglynn J. C. Dankwort T. Kienle L. Bandeira N. A. G. Fraser J. P. Gibson E. K. (2019). The rapid electrochemical activation of MoTe_2_ for the hydrogen evolution reaction. Nat. Commun. 10, 4916. 10.1038/s41467-019-12831-0 31664018 PMC6820771

[B32] Qian X. Liu J. Fu L. Li J. (2014). Quantum spin Hall effect in two-dimensional transition metal dichalcogenides. Science 346, 1344–1347. 10.1126/science.1256815 25504715

[B33] Qiao H. Huang Z. Liu S. Liu Y. Li J. Qi X. (2018). Liquid-exfoliated molybdenum telluride nanosheets with superior electrocatalytic hydrogen evolution performances. Ceram. Int. 44, 21205–21209. 10.1016/j.ceramint.2018.08.166

[B34] Singh S. Deb J. Sarkar U. Sharma S. (2022a). MoSe_2_/multiwalled carbon nanotube composite for ammonia sensing in natural humid environment. J. Hazard. Mater. 435, 128821. 10.1016/j.jhazmat.2022.128821 35468389

[B35] Singh S. Deb J. Singh J. V. Sarkar U. Sharma S. (2022b). Highly selective ethyl mercaptan sensing using a MoSe_2_/SnO_2_ composite at room temperature. ACS Appl. Mat. Interfaces 14, 23916–23927. 10.1021/acsami.1c25112 35548976

[B36] Singh S. Kim J. Rabe K. M. Vanderbilt D. (2020). Engineering weyl phases and nonlinear Hall effects in t_d_-MoTe_2_ . Phys. Rev. Lett. 125, 046402. 10.1103/physrevlett.125.046402 32794815

[B37] Sun Y. Felser C. Yan B. (2015). Graphene-like Dirac states and quantum spin Hall insulators in square-octagonal *MX* _2_ (*M* = Mo, W; *X* = S, Se, Te) isomers. Phys. Rev. B 92, 165421. 10.1103/physrevb.92.165421

[B38] Sun Y. Wang Y. Sun D. Carvalho B. R. Read C. G. Lee C.-H. (2016). Low-temperature solution synthesis of few-layer 1T ′-MoTe_2_ nanostructures exhibiting lattice compression. Angew. Chem. Int. Ed. Engl. 55, 2880–2884. 10.1002/ange.201510029 26804980

[B39] Tan C. Cao X. Wu X.-J. He Q. Yang J. Zhang X. (2017). Recent advances in ultrathin two-dimensional nanomaterials. Chem. Rev. 117, 6225–6331. 10.1021/acs.chemrev.6b00558 28306244

[B40] Wang Q. H. Kalantar-Zadeh K. Kis A. Coleman J. N. Strano M. S. (2012). Electronics and optoelectronics of two-dimensional transition metal dichalcogenides. Nat. Nanotechnol. 7, 699–712. 10.1038/nnano.2012.193 23132225

[B41] Wang Y. Shen Y. Xiao X. Dai L. Yao S. An C. (2021). Topology conversion of 1T MoS_2_ to S-doped 2H-MoTe_2_ nanosheets with Te vacancies for enhanced electrocatalytic hydrogen evolution. Sci. China Mat. 64, 2202–2211. 10.1007/s40843-020-1612-y

[B42] Watzele S. Fichtner J. Garlyyev B. Schw Mmlein J. N. Bandarenka A. S. (2018). On the dominating mechanism of the hydrogen evolution reaction at polycrystalline Pt electrodes in acidic media. ACS Catal. 8, 9456–9462. 10.1021/acscatal.8b03365

[B43] Xi G. Liu Y. Wang X. Liu X. Peng Y. Qian Y. (2006). Large-scale synthesis, growth mechanism, and photoluminescence of ultrathin Te nanowires. Cryst. Growth & Des. 6, 2567–2570. 10.1021/cg0603218

[B44] Xu M. Liang T. Shi M. Chen H. (2013). Graphene-like two-dimensional materials. Chem. Rev. 113, 3766–3798. 10.1021/cr300263a 23286380

[B45] Zazpe R. Sopha H. Charvot J. Krumpolec R. Rodriguez-Pereira J. Michalička J. (2021). 2D MoTe_2_ nanosheets by atomic layer deposition: Excellent photo- electrocatalytic properties. Appl. Mater. Today 23, 101017. 10.1016/j.apmt.2021.101017

[B46] Zhang G. Liu H. Qu J. Li J. (2016). Two-dimensional layered MoS_2_: Rational design, properties and electrochemical applications. Energy Environ. Sci. 9, 1190–1209. 10.1039/c5ee03761a

[B47] Zhang X. Jia F. Song S. (2021). Recent advances in structural engineering of molybdenum disulfide for electrocatalytic hydrogen evolution reaction. Chem. Eng. J. 405, 127013. 10.1016/j.cej.2020.127013

