# Supplementary material for: Facile hydrothermal synthesis of layered 1T′ MoTe2 nanotubes as robust hydrogen evolution electrocatalysts
Source: Front Chem. 2022 Sep 27;10:1005782. doi: 10.3389/fchem.2022.1005782 (PMC9551219; doi:10.3389/fchem.2022.1005782)
Supplement: Supplementary file 1 [file DataSheet1.docx]

# Supplementary Material

## Facile hydrothermal synthesis of layered 1T' MoTe_2_ nanotubes as robust hydrogen evolution electrocatalysts

Yuxi Lei^a, b*^, Xuefeng Xiao^a, b^, Tianpeng Ma^a, b^, Weiyin Li^a, b^, Huan Zhang^a, b^, Chao Ma^a, b^

^a^School of Electrical & Information Engineering, North Minzu University, Yinchuan, 750021, China

^b^The Key Laboratory of Physics and Photoelectric Information Functional Materials, North Minzu University, Yinchuan, 750021, China

Corresponding author. E-mail address: Lei_yuxi@nun.edu.cn (Y. X. Lei).


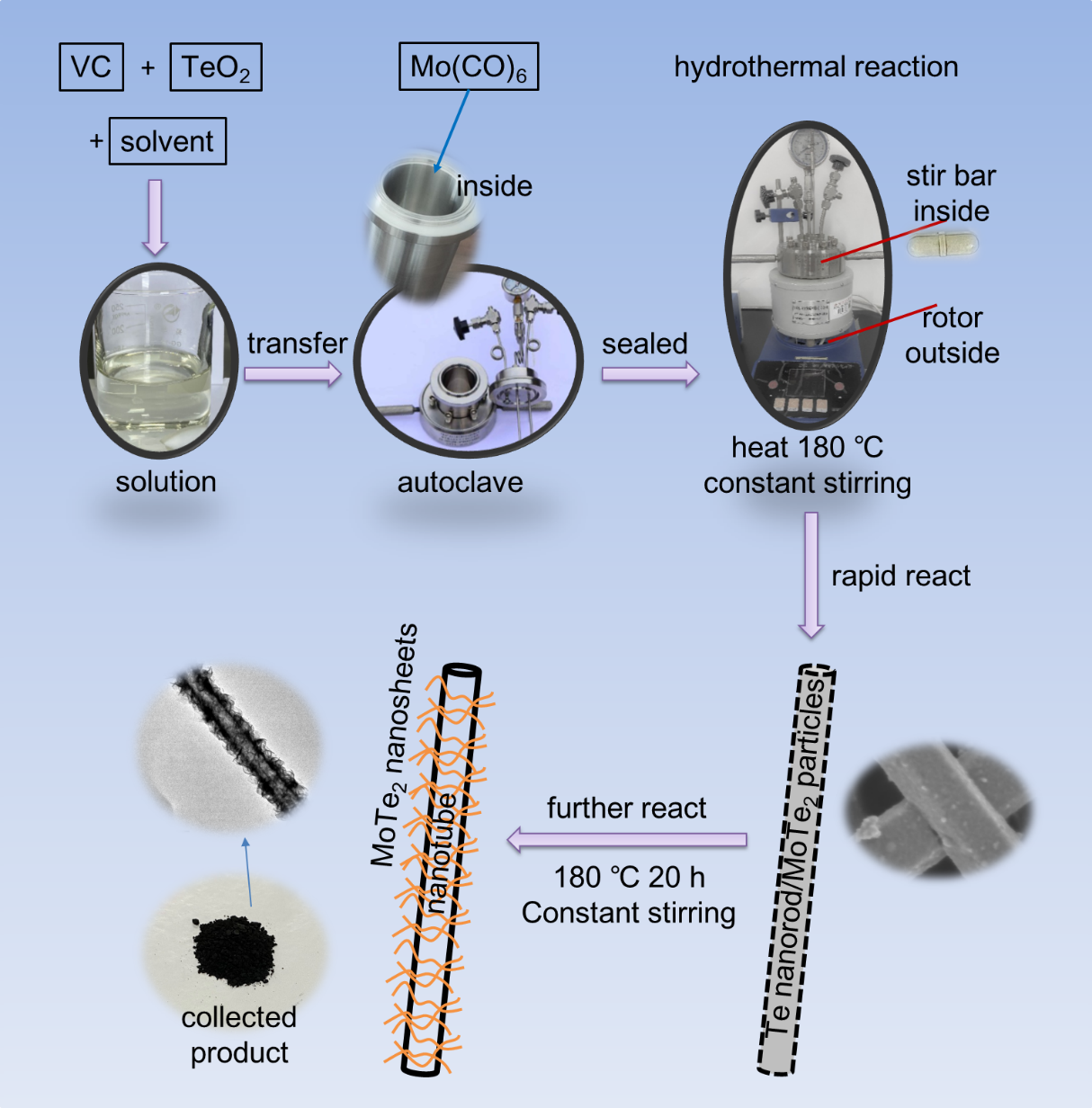


**Figure S1**. Schematic illustration of one-step hydrothermal method for synthesizing MoTe_2_ nanotubes.

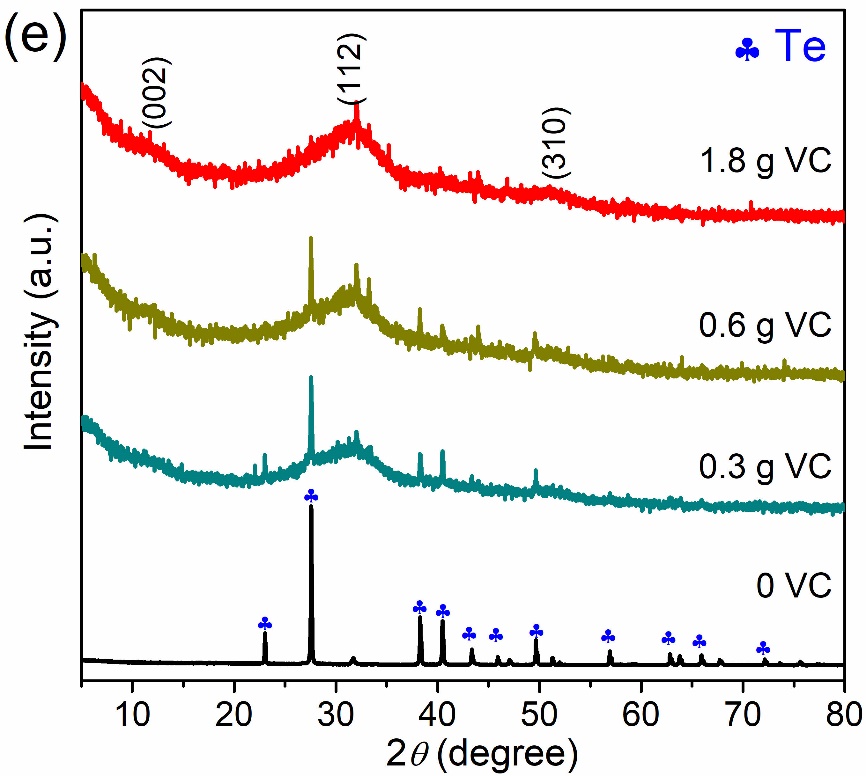


**Figure S2**. SEM images of MoTe_2_ products synthesized with different amount of ascorbic acid (VC): (a) 0.0 g, (b) 0.3 g, (c) 0.6 g and (d) 1.8 g. (e) The corresponding XRD patterns.


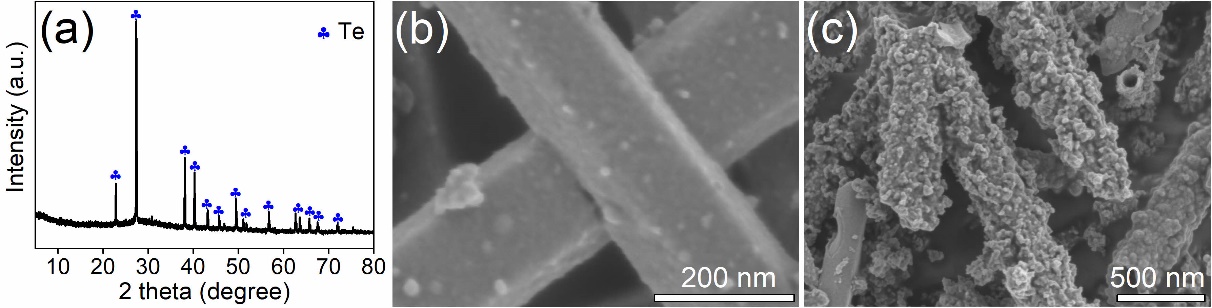


**Figure S3**. XRD pattern (a) and SEM images (b, c) of MoTe_2_ nanotubes synthesized by one-step hydrothermal process at 3 h reaction with 1.2 g of ascorbic acid (VC).

The growth of MoTe_2_ nanotubes was explored using controlled experiments under different conditions. The VC additive plays a key role in the formation of uniform tubular MoTe_2_ nanosheets. A series of experiments were conducted with different amounts of ascorbic acid, while keeping the other experimental conditions the same. Nonuniform Te (main component) micro-rods and irregular blocks were obtained in the absence of ascorbic acid [Figure S2(a)]. When a small quantity (0.3 g) of ascorbic acid was used, MoTe_2_ nanotubes with a diameter of approximately 500 nm [Figure S2(b)] were formed; however, the nanotubes were composed of numerous small particles with rough surfaces. When the amount of ascorbic acid was increased to 0.6 g, uniform MoTe_2_ nanotubes without nanosheets were obtained [Figure S2(c)]. When the amount of ascorbic acid was further increased to 1.2 g, uniform MoTe_2_ nanotubes decorated with few-layer sheets were formally formed (text section). The morphology of the products changed slightly when the amount of ascorbic acid was increased further [Figure S2(d)]. Thus, the additive contributes to the formation of sheet-like MoTe_2_ nanotubes, and the optimal amount of ascorbic acid for the synthesis of uniform MoTe_2_ nanotubes is above 1.2 g. Additionally, Te impurities remain in the product synthesized at insufficient VC [Figure S2(e)].

Furthermore, VC can rapidly reduce TeO_2_ to Te nanorods in the precursor solution and act as a template/seed to grow MoTe_2_ nanosheets. In the sample hydrothermally treated for 3 h, large quantity of Te/MoTe_2_ nanotubes were initially formed, which further grew into integrated MoTe_2_ nanotubes (Figure. S3). Then, few-layer sheets MoTe_2_ nanotubes were uniformly formed under a long reaction time and an appropriate amount of VC.


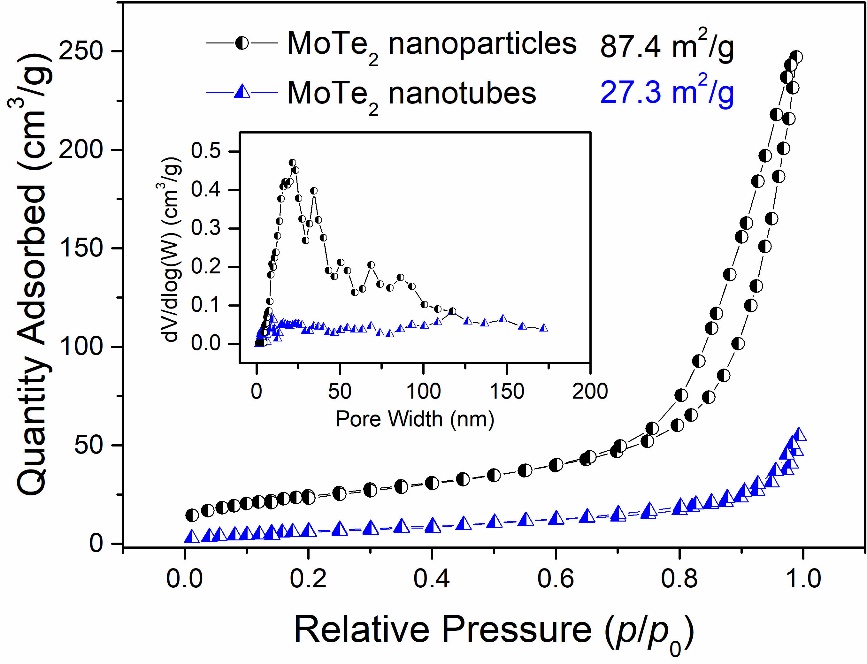


**Figure S4**. (a) N_2_ adsorption-desorption isotherms of as prepared MoTe_2_ nanocrystals. The insets show the corresponding pore size curves and BET surface area.


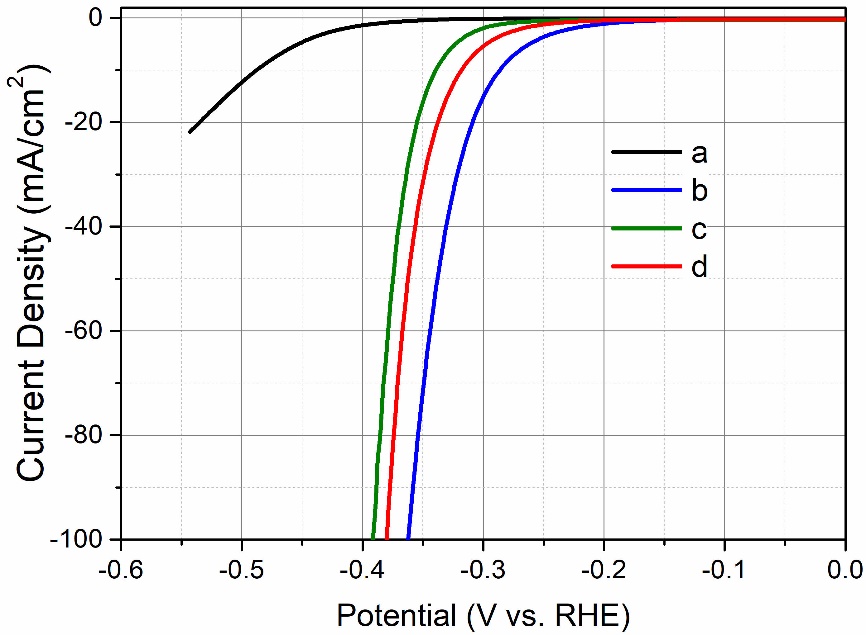


**Figure S5**. LSV curves of other MoTe_2_ nanotubes obtained by different amount of VC (a) 0g, (b) 0.3 g, (c) 0.6 g and (d) 1.8 g.

**Figure S6**. Characterization of MoTe_2_ nanotubes after long-term HER test. Low-resolution (a) and high-resolution SEM images (b) after 20 h chronoamperometry test in 0.5 M H_2_SO_4_; High-resolution XPS spectra of Te 3d (c) and Mo 3d (d) before and after 20 h chronoamperometry test in 0.5 M H_2_SO_4_. The catalyst was loaded on square glass carbon plates for characterization.

**Table S1** Comparison of HER properties of MoTe_2_ related electrocatalysts

| Electrocatalysts | Synthesis method | @*η*_10_ (mV) | @*η*_100_ (mV) | Tafel slope  (mV∙dec^−1^) | electrolyte | Ref. |
| --- | --- | --- | --- | --- | --- | --- |
| 1T'-MoTe_2_ | Flux method | 356 | - | 127 | 0.5 M H_2_SO_4_ | [[1](#_ENREF_1)] |
| 1T'-MoTe_2_/CC | CVD | 231 | ~ 420 | 127 | 0.5 M H_2_SO_4_ | [[2](#_ENREF_2)] |
| MoTe_2_ | Chemical exfoliation | 380 | - | 57 | 0.5 M H_2_SO_4_ | [[3](#_ENREF_3)] |
| 1T'-MoTe_2_ | Solid-state method | 340 | - | 78 | 1.0 M H_2_SO_4_ | [[4](#_ENREF_4)] |
| MoTe_2_ nanosheets | Liquid exfoliation | 309 | - | 119 | 0.5 M H_2_SO_4_ | [[5](#_ENREF_5)] |
| MoTe_2_ nanotubes | Hydrothermal method | 283 | ~ 500 | 102 | 0.5 M H_2_SO_4_ | [[6](#_ENREF_6)] |
| Pt/1T'-MoTe_2_ | Electrochemical deposition | 23 | - | 22 | 0.5 M H_2_SO_4_ | [[7](#_ENREF_7)] |
| 1T'-MoTe_2_/Ni foam | Chemical exfoliation | 82 | - | 50 | 0.5 M H_2_SO_4_ | [[8](#_ENREF_8)] |
| MoTe_2_ nanowire | Nanocasting strategy | 410 | - | 58 | 0.5 M H_2_SO_4_ | [[9](#_ENREF_9)] |
| MoTe_2_/TiO_2_ nanotubes | ALD | 373 | - | 108 | 0.5 M H_2_SO_4_ | [[10](#_ENREF_10)] |
| Nanosheets decorated MoTe_2_ nanotubes | Hydrothermal method | 317 | 349 | 54 | 0.5 M H_2_SO_4_ | This work |

References

[1] Jinbong, S.;Jun-Ho, L.;Suyeon, C.;Byungdo, J.;Hyo Won, K.;Min, K.;Dohyun, K.;Young-Min, K.;Sang Ho, O.;Sung Wng, K.;Young Hee, L.;Young-Woo, S.; Heejun, Y. Active hydrogen evolution through lattice distortion in metallic MoTe_2_. *2D Materials* **2017**, *4*, 025061.

[2] Lu, D.;Ren, X.;Ren, L.;Xue, W.;Liu, S.;Liu, Y.;Chen, Q.;Qi, X.; Zhong, J. Direct Vapor Deposition Growth of 1T′ MoTe_2_ on Carbon Cloth for Electrocatalytic Hydrogen Evolution. *ACS Applied Energy Materials* **2020**, *3*, 3212-3219.

[3] Luxa, J.;Vosecký, P.;Mazánek, V.;Sedmidubský, D.;Pumera, M.;Lazar, P.; Sofer, Z. Layered Transition-Metal Ditellurides in Electrocatalytic Applications—Contrasting Properties. *ACS Catalysis* **2017**, *7*, 5706-5716.

[4] McGlynn, J. C.;Cascallana-Matías, I.;Fraser, J. P.;Roger, I.;McAllister, J.;Miras, H. N.;Symes, M. D.; Ganin, A. Y. Molybdenum Ditelluride Rendered into an Efficient and Stable Electrocatalyst for the Hydrogen Evolution Reaction by Polymorphic Control. *Energy Technology* **2018**, *6*, 345-350.

[5] Qiao, H.;Huang, Z.;Liu, S.;Liu, Y.;Li, J.; Qi, X. Liquid-exfoliated molybdenum telluride nanosheets with superior electrocatalytic hydrogen evolution performances. *Ceramics International* **2018**, *44*, 21205-21209.

[6] Bhat, K. S.; Nagaraja, H. S. Performance evaluation of molybdenum dichalcogenide (MoX2; X= S, Se, Te) nanostructures for hydrogen evolution reaction. *International Journal of Hydrogen Energy* **2019**, *44*, 17878-17886.

[7] Seok, J.;Lee, J.-H.;Bae, D.;Ji, B.;Son, Y.-W.;Lee, Y. H.;Yang, H.;Cho, S.; J., P. Hybrid catalyst with monoclinic MoTe2 and platinum for efficient hydrogen evolution

Applications of 2D MXenes in energy conversion and storage systems. *APL Materials* **2019**, *7*, 071118.

[8] He, Y.;Boubeche, M.;Zhou, Y.;Yan, D.;Zeng, L.;Wang, X.;Yan, K.; Luo, H. Topologically nontrivial 1T’-MoTe_2_ as highly efficient hydrogen evolution electrocatalyst. *Journal of Physics: Materials* **2020**, *4*, 014001.

[9] Mao, J.;Zhou, L.;Li, Y.;Tao, Y.;Chai, K.;Shi, Y.; Xu, W. Synthesis of MoTe2 nanowire as an efficient hydrogen evolution reaction material. *Materials Letters* **2021**, *290*, 129471.

[10] Zazpe, R.;Sopha, H.;Charvot, J.;Krumpolec, R.;Rodriguez-Pereira, J.;Michalička, J.;Mistrík, J.;Bača, D.;Motola, M.;Bureš, F.; Macak, J. M. 2D MoTe_2_ nanosheets by atomic layer deposition: Excellent photo- electrocatalytic properties. *Applied Materials Today* **2021**, *23*, 101017.
